# Gorham's disease involving the left parietal bone: a case report

**DOI:** 10.1186/1757-1626-1-258

**Published:** 2008-10-22

**Authors:** Vijay Parihar, Yad Ram Yadav, Dhananjaya Sharma

**Affiliations:** 1 Assistant Professor Neurosurgery, Neurosurgery Unit, Department of General Surgery, NSCB Govt. Medical College & Hospital, Jabalpur, MP, 482002, India

## Abstract

**Introduction:**

Gorham's disease is a rare bone disease, is characterized by the proliferation of thin-walled vascular channels associated with regional osteolysis. There have been fewer than 150 cases reported in the literature. Shoulder and pelvic region is common site. Skull is the least common site of involvement.

**Case Presentation:**

We describe a case of a 35-year-old female of Indian origin presented with this rare condition involving her left parietal bone. She was treated successfully with excision of diseased bone and cranioplasty.

**Conclusion:**

This is the first reported case in India. We provide a review of the clinical, radiological and pathological diagnosis of this rare condition and describe treatment options.

## Introduction

Gorham disease (massive osteolysis of Gorham, vanishing or disappearing bone disease, Gorham-Stout syndrome and phantom bone disease) is a rare disorder characterized by a nonfamiliar, histologically benign vascular proliferation originating in bone and producing progressive resorption of all or a portion of the bone [1]. This uncommon condition occurs sporadically and is most often observed in children and young adults of either sex. Involvement of almost every bone has been reported, although there is a predilection for bones that develop by intramembranous ossification, with the shoulder girdle and mandible being the most common bones affected. The lesion is typically nonexpansile and nonulcerative and is usually monocentric but locally aggressive, with resorption of the affected bone. The vascular lesion may spread into soft tissue and contiguous bones [1,2]. The pathogenesis of Gorham-Stout disease remains unknown, although it has been suggested that there is an increase in the sensitivity of osteoclast precursors to humoral factors, which promote osteoclast formation and bone resorption and operate at the level of the bone microenvironment [3].

## Case presentation

**Figure 1:**
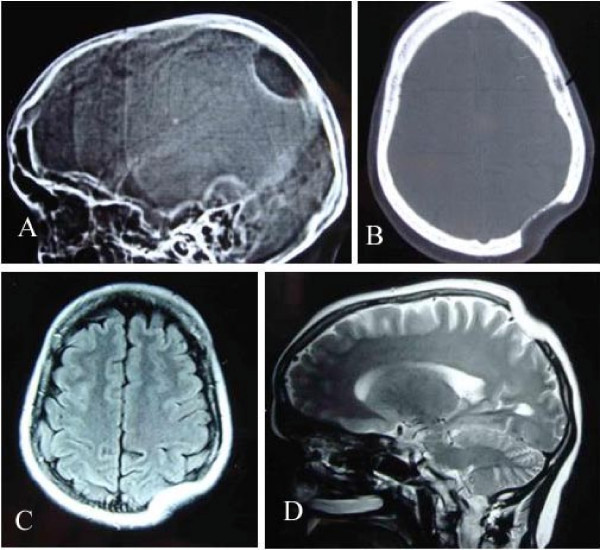
**X-ray skull lateral view (A) showing a osteolytic area in left parietal region.** CT scan bony window (B), MRI T1W Axial (C) and T2W Sagittal (D) revealing skull defect with normal brain parenchyma.

**Figure 2:**
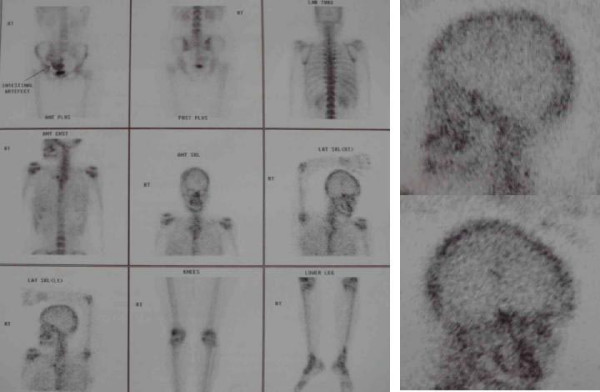
Tc-99 m MDP bone scan reveals no bone uptake in the resorbed site and around the margin of defect.

A 35-year-old female of Indian origin presented with complaint of depression over left parietal area noticed accidentally about four years back. At that time the size is about a coin. It is slowly and gradually progressive in nature. At present it is about 4 × 4 cm in size. There were no other symptoms at this time duration. There was no history of any trauma. Her neurological examination was normal. Local examination shows a well-defined nontender rounded left parietal depression of 4 × 4 cm in size without any soft tissue mass with normal overlying scalp. Plain x-ray skull was showing a well-demarcated rounded lytic area in parietal region with normal margin. (Figure 1A) There is no other abnormality seen in plain x-ray. CT scan revealed a well-defined skull defect with complete disappearance of the central bone matrix and no evidence of new bone formation (Figure 1B). The soft tissue widows showed no associated soft tissue mass in or around the calvarial defect. MRI brain showing calvarial defect to be filled with thin soft tissues that were hypointense on both T1-weighted and T2-weighted (Figure 1C&1D). Tc-99 m MDP bone scintigraphy reveals no bone uptake in the resorbed site as well as around the margin. Whole-body bone scan was undertaken, which revealed no similar bony lesion any where in the body. (Figure 2). The preoperative differential diagnosis included Paget disease at destructive stage, eosinophilic granuloma, Brown tumor, and osteolytic metastasis but none of them correlated well with the clinical and radiologic features.

**Figure 3:**
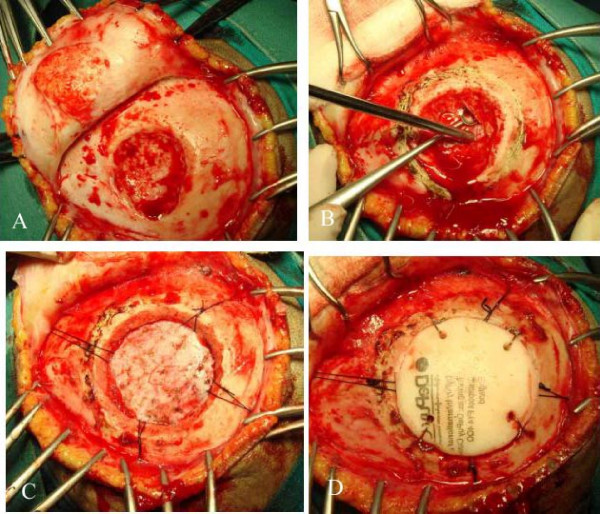
Operative pictures showing left parietal flap (A), removal of inner table (B), normal underlying dura (C), and cranioplasty with bone cement (D).

**Figure 4:**
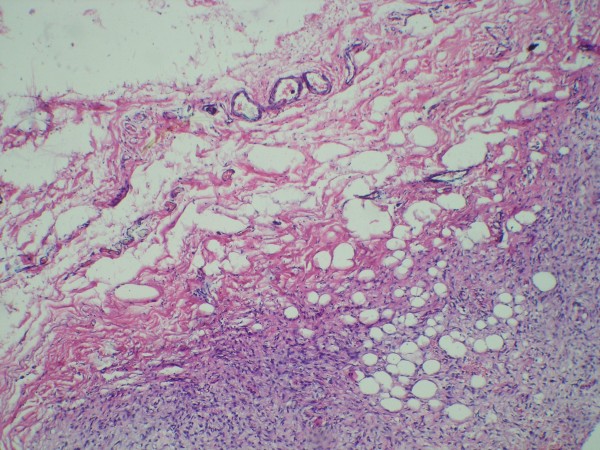
Photomicrograph of the bone specimen shows fibromyxoid tissue with highly vascularised collagenous tissue and the small capillary-like vessels.

A left parietal craniectomy was performed, a bony defect of 4 × 4 cm with a very thin remainder of the inner table was found. There is involvement of overlying galea and subgaleal tissue in form of thickening and increased vascularity was found. (Figure 3A) The inner table was removed and bone specimens taken from the edges. (Figure 3B) Dura was normal in texture colour and vascularity.(Figure 3C) Cranioplasty done with bone cement. (CMW-1 Johnson's and Johnson's) (Figure 3D). Histopathological examination of bone pieces showed intraosseous angiomatosis with mixed patterns of bone destruction, fibrous connective tissue replacement by highly vascularised collagenous tissue, and abnormal small vessel proliferation (Figure 4). The combination of clinical, imaging, and pathologic findings strongly suggested the diagnosis as Gorham disease. The patient had an uneventful postoperative course and was discharged on 7^th ^postoperative day.

## Discussion

Jackson first reported a case of massive osteolysis of the humerus in a 12-year-old boy [4]. In 1955, Gorham and Stout further characterized the main pathologic features of this rare disease as nonmalignant intraosseous proliferation of hemangiomatous or lymphangiomatous tissue that caused massive osteolysis [5]. Gorham disease is a nonhereditary disease with no sex predilection. Most patients are younger than 40 years. More than 150 cases have been documented in the literature [6]. The bone of pelvic and shoulder regions were most frequently involved, although any bone may be affected. The skull is among the least common site [6-8]. To the best of our knowledge this is the first reported case of Gorham's disease from India.

The proposed pathogenesis of Gorham's disease is nonmalignant, neoplastic proliferation of hemangiomatous or lymphangiomatous tissue [6-8]. Some authors have suggested local hypoxia and acidic environment, and some hydrolytic enzymes such as acid phosphatase and leucine aminopeptidase can cause the bone destruction [7-9]. Others have also postulated the role of mechanical forces may promote bone resorption and that trauma might triggers the process [10]. Johnson and McClure suggested that there are two stages of Gorham disease [4]. The first stage of hemangiomatosis characterized by vascular proliferation in connective tissue. This explains some of the pathology reports of Gorham disease as "skeletal hemangioma" [6,7,10]. Second is the stage of fibrosis that replaces the absorbed bone. Whether osteoclasts are involved in the mechanism of bone destruction remains controversial. Most authors have not observed osteoclasts in the areas of excessive bone resorption by microscopy [7,9,10].

Clinical manifestations include pain, swelling, or a pathologic fracture, whereas others may be asymptomatic or have an insidious onset of soft tissue atrophy [6-9]. There are no specific laboratory findings [9,10]. Preoperative Gorham disease must be distinguished from osteolysis secondary to other pathologic processes, including the hereditary, metabolic, neoplastic, infectious, and immunologic etiologies [6,8]. Common differential diagnoses include hereditary multicentric osteolysis, essential osteolysis with nephropathy, metastasis, osteomyelitis, and rheumatoid arthritis [6]. The clinical findings are usually helpful in ruling out these diseases.

The osseous deformity or pathologic fracture in Gorham disease is common, but more serious complications are infrequent [6,8,9]. Paraplegia may occur in cases of vertebral involvement [10]. Many different kinds of therapy have been attempted. A complex treatment of hormones with calcium salts and vitamins showed no efficacy. Radiation therapy has been reported to shift the disease from active to inactive phase or achieve pain relief with varying degrees of success [6,8]. Most authors have suggested that surgical resection with artificial bone replacement has been the most effective treatment for disease control, as the homologous bone grafts may also undergo osteolysis [6,8].

The initial radiologic feature of Gorham disease may reveal radiolucent foci in the intramedullary or subcortical regions that resemble osteoporosis [6,10]. Subsequently, progressive dissolution and disappearance of a portion of the bone may occur. The osteolysis may extend to the contiguous bone and cross the intervening joint [6,8,10]. The osseous destruction may persist for a period of years and may eventually stabilize [7,10]. Tc-99 m bone scintigraphy may demonstrate increased uptake of the radiopharmaceutical agents on the initial images and, subsequently, an area of decreased uptake corresponding to the diminished bone region [7,10].

The decreased uptake over the left parietal area seen in our case may indicate decreased in bone in that region. CT with bone window setting provides delineation of the extent of osteolysis, and 3D imaging is useful for surgical planning for skull reconstruction. MR imaging, however, allows superior delineation of bone marrow and soft tissue involvement. Although other pathologic conditions in the skull such as juvenile Paget disease, eosinophilic granuloma, Brown tumor, and osteolytic metastases may have similar imaging findings, the long asymptomatic clinical course and typical CT and MR imaging findings may allow differentiation of Gorham disease.

## Conclusion

The diagnosis of Gorham's disease is one of exclusion, based mainly on clinico-radiological findings, evolution and compatible histological findings. The aim of this case report is to emphasize the Gorham's disease as a rare differential diagnosis for skull lesions.

## Consent

Written informed consent was obtained from the patient for publication of this case report and accompanying images. Patient has given their informed consent for the case report to be published. The copies of the consent documentation available with corresponding author on request at any time.

## Competing interests

The authors declare that they have no competing interests.

## Authors' contributions

PV analyzed and interpreted the patient data regarding the Gorhams disease and performed the surgical procedure. YYR was a major contributor in writing the manuscript. SD made correction and approved the final manuscript.
